# Using machine learning on cardiorespiratory fitness data for predicting hypertension: The Henry Ford ExercIse Testing (FIT) Project

**DOI:** 10.1371/journal.pone.0195344

**Published:** 2018-04-18

**Authors:** Sherif Sakr, Radwa Elshawi, Amjad Ahmed, Waqas T. Qureshi, Clinton Brawner, Steven Keteyian, Michael J. Blaha, Mouaz H. Al-Mallah

**Affiliations:** 1 King Saud bin Abdulaziz University for Health Sciences, Riyadh, Saudi Arabia; 2 King Abdullah International Medical Research Center, Riyadh, Saudia Arabia; 3 Heart and Vascular Institute, Henry Ford Hospital System, Detroit, MI, United States of America; 4 Princess Nourah bint Abdulrahman University, Riyadh, Saudi Arabia; 5 Wake Forest School of Medicine, Medical Center Boulevard, Winston-Salem, NC, United States of America; 6 Johns Hopkins Medicine, Baltimore, Maryland, United States of America; 7 University of Taru, Taru, Estonia; Northeast Normal University, CHINA

## Abstract

This study evaluates and compares the performance of different machine learning techniques on predicting the individuals at risk of developing hypertension, and who are likely to benefit most from interventions, using the cardiorespiratory fitness data. The dataset of this study contains information of 23,095 patients who underwent clinician- referred exercise treadmill stress testing at Henry Ford Health Systems between 1991 and 2009 and had a complete 10-year follow-up. The variables of the dataset include information on vital signs, diagnosis and clinical laboratory measurements. Six machine learning techniques were investigated: LogitBoost (LB), Bayesian Network classifier (BN), Locally Weighted Naive Bayes (LWB), Artificial Neural Network (ANN), Support Vector Machine (SVM) and Random Tree Forest (RTF). Using different validation methods, the RTF model has shown the best performance (AUC = 0.93) and outperformed all other machine learning techniques examined in this study. The results have also shown that it is critical to carefully explore and evaluate the performance of the machine learning models using various model evaluation methods as the prediction accuracy can significantly differ.

## Introduction

Hypertension is a major condition that can lead to many severe illnesses such as stroke and heart disease [1]. Risk assessment of the disease is significantly complicated and depends on many factors and environmental conditions that can significantly raise blood pressure readings. According to the World Health Organization (WHO), high blood pressure causes one in every eight deaths and therefore Hypertension is considered the third leading killer in the world [2]. There are around a billion of hypertensive patients around the word and around four million patients die every year. In the Middle Eastern region, cardiovascular disease and stroke are the main cause of death and illness. They resulted in 31% of deaths and currently hypertension affects around 26% of the adult population in the region [3–6]. Currently there is no clear causes for high blood pressure however there are several factors and conditions may play an important role in its development such as smoking, obesity, lack of physical activity, salty diet, stress, age, family history, chronic kidney disease and thyroid disorders. The main goal of intervention is to reduce blood pressure and control the heart disease.

The Henry Ford Exercise Testing (FIT) Project [7] is a retrospective cohort that includes the information of 69,981 patients who had undergone physician referred treadmill stress testing at Henry Ford Hospital System in Detroit, MI from January 1, 1991- May 28, 2009. Briefly, the study population was limited to patients who are over the age of 18 years of age at the time of stress testing and excluded patients undergoing modified or non-Bruce protocol [8] stress tests. Information regarding the patient’s medical history, demographics, medications, cardiovascular disease risk factors were obtained at the time the tests were done by nurses and exercise physiologists, as well as searches through the electronic medical records. All study patients underwent clinically indicated treadmill stress testing utilizing the standard Bruce Protocol. All stress tests were performed in accordance with standard American College of Cardiology/American Heart Association Guidelines [9]. For the full details of The FIT Project, we refer the reader to [7]. Several studies [10–13] have used conventional statistical techniques to predict various medical outcomes using the FIT project data.

Machine learning (ML) [14, 15] is a modern data analysis technique with the unique ability to learn and improve its performance without being explicitly programmed and without human instruction. The main goal of supervised machine learning classification algorithms [16] is to explain the dependent variable in terms of the independent variables. The algorithms get adjusted based on the training sample and an error signal. ML algorithms automatically scan and analyze all predictor variables in a way that prevents overlooking any potentially important predictor variables even if it was unexpected. Therefore, ML is acknowledged as a powerful tool which dramatically changes accessibility of science, research and practice in all domains [17]. Medicine and Healthcare are no different [18–21]. In this study, we investigate and compare the performance of several machine learning techniques that use cardiorespiratory fitness data for predicting individuals at risk of developing hypertension who are most likely to benefit from interventions. We evaluate and compare six well-known machine learning techniques to come up with the best model to predict the risk of hypertension. The models have been evaluated using different metrics. The machine learning techniques used in this study are: *LogitBoost* (LB), *Bayesian Network* classifier (BN), *Locally Weighted Naive Bayes* (LWB), *Artificial Neural Network* (ANN), *Support Vector Machine* (SVM) and *Random Tree Forest* (RTF). Different validation techniques and evaluation metrics are compared and reported.

## Materials and methods

### Henry Ford FIT dataset

The dataset was collected from patients who underwent treadmill stress testing by physician referrals at Henry Ford Affiliated Hospitals in metropolitan Detroit, MI in the U.S. The FIT Project data has been obtained from the electronic medical records, administrative databases, and the linked claim files and death registry of the hospital [7]. Study participants underwent routine clinical treadmill exercise stress testing using the standard Bruce protocol between January 1st, 1991 and May 28th, 2009. The day the treadmill test was performed served as the baseline for this study. The exercise stress test would be terminated by the supervising clinician if the patient had exercise-limiting chest pain, shortness of breath, or other limiting symptoms independent of the achieved heart rate. Furthermore, testing could also be terminated early at the discretion of the supervising clinician for significant arrhythmias, abnormal hemodynamic responses, diagnostic ST-segment changes, or if the participant was unwilling or unable to continue [22].

The total number of patients included in this study is (n = 23,095). The data set includes 43 attributes containing information on vital signs, diagnosis and clinical laboratory measurements. The baseline characteristics of the included cohort are shown in Table 1. The data set contains 23, 095 individuals (12,694 males (55%) and 10,401 (45%) females) with ages that range between17 and 96. Half of the patients have a family history of cardiovascular diseases. During the 10-years follow-up, around 35% of the patients experienced hypertension. Male hypertension patients represent around 55% of the total hypertension patients while female patients represent around 44% of the total hypertension patients.

**Table 1:** Dataset description.

**Age (yrs +/- SD)**	49 +/- 12
**Gender**	
**Male**	12,694 (55%)
**Female**	10,401 (45%)
**Race**	
**Black**	4694 (20%)
**Other**	18401 (80%)
**Reason for Test**	
**Chest Pain**	12581 (54%)
**Shortness of Breath**	1956 (8%)
**Pre-Operation**	255 (1%)
**Known Coronary Artery Disease**	524 (2%)
**Rule out Ischemia**	2286 (10%)
**Abnormal prior test**	1004 (4%)
**Stress**	
**Peak METS (Mean +/- SD)**	10.2 +/- 2.79
**Resting Systolic Blood Pressure (Mean +/- SD)**	124 +/- 17
**Resting Diastolic Blood Pressure (Mean +/- SD)**	79 +/- 10
**Resting Heart rate (Mean +/- SD) beat per minute (bpm)**	73 +/- 12
**Peak Diastolic Blood Pressure (Mean +/- SD)**	82 +/- 13
**Peak Heart Rate (Mean +/- SD) beat per minute (bpm)**	159 +/- 17
**Past Medical History**	
**Diabetes**	1887 (8%)
**History of Smoking**	9,518 (41%)
**Family History**	11,865 (51%)
**History of Hyperlipidemia**	7,769 (34%)
**History of Coronary Artery Bypass Graft**	314 (1%)

### Data preprocessing

One of the main steps that affects the performance and quality of prediction of machine learning models is data quality and data preprocessing. Data preprocessing includes handling missing values, smooth noisy data, identify or remove outliers, normalization, transformation, etc. Therefore, several steps have been applied to handle some issues on the dataset.

- **Outliers**: a value of an attribute is considered as an outlier if it deviates from the expected value for this attribute. Outliers has been handled using inter-quartile range (*IQR*). The *IQR* identifies outliers in the dataset by identifying over ranging data in data. The *IQR* is a good choice for handling the outliers since the dataset used in this study is nearly symmetric means that its median equals its midrange. The *IQR* is evaluated as *IQR* = *Q*3 − *Q*1 where *Q*3 and *Q*1 are the upper and lower quartiles, respectively. Outliers are records that fall below *Q*1 − (1.5 * *IQR*) or above *Q*3 + (1.5 * *IQR*). The number of records identified as outliers or extreme values and has been removed in the dataset used in this work is 192 records.
- **Missing values**: The only attribute that has missing values is Peak Diastolic blood pressure and the number of individuals with missing Peak Diastolic blood pressure is 72. All the values for this attribute are replaced by the mean value of this attribute.
- **Discretization**: Aims to reduce the number of values for continuous attributes. This is done by splitting the range of the continuous attribute into intervals. Discretization reduces the time needed to build the prediction model and improve the prediction results [23]. The following attributes have been discretized: Age, METS, Resting Systolic blood pressure, Resting Diastolic blood pressure, The Percentage of Heart Rate Achieved, Peak Heart Rate and Peak Diastolic blood pressure.
- **Sampling**: The dataset used in this study consists of 23,095 with 8,090 patients with experienced hypertension and the rest did not. The most common metric used to evaluate machine learning techniques is accuracy. This measure does not work properly when the data is imbalanced (the variance between patients who experienced hypertension and those who did not experience hypertension is considerably high). However, the nature of our prediction problem requires a high rate of correct detection of patients who are at high risk of developing hypertension. In general, there are two different method to address the imbalanced dataset and obtain a balanced dataset (the number of patients who experienced hypertension is close to the number of patients who did not experienced hypertension). The first method is over-sampling the minority class (patients who experienced hypertension) [24] and the second method is under-sampling the majority class [25]. In this study, we used both under-sampling and over-sampling to handle the imbalanced data problem and compare the performance of both techniques. We used the Synthetic Minority Over-sampling (SMOTE) Technique [26]. It is an over-sampling techniques in which the minority class is over-sampled by creating “synthetic” examples rather than by over-sampling with replacement. SMOTE selects the minority class samples (patients experienced hypertension) and creates “synthetic” samples along the same line segment joining some or all k nearest neighbors belonging to the minority class. More precisely, the over-sampling is done as follows:
  - Take sample of the dataset and find its nearest neighbors.
  - To create a synthetic data point, take the vector between a one of the data points *P* in the sample dataset and one of *P* k-nearest neighbors.
  - Multiply this vector by a random number *x* which lies between 0 and 1.
  - Add this to *P* to create the new synthetic data point.
  The percentage of SMOTE instances created in our experiment is 30% (2,427 records from the minority class). In addition, we used the spread Sub-sample instance method as an under-sampling technique [25]. The spread sub-sample method outputs a random sub-sample of a dataset. This instance method allows you to mention the maximum “spread” between the minority and majority classes. You may specify that there is at most 2:1 difference in the frequency of the majority and minority classes. In this study, we used this method to maintain equal ratio between the majority and minority classes.

### Feature selection

Feature selection is an essential part of building a good prediction model for many reasons [27]. For example, it implies some degree of cardinality reduction by reducing the number of attributes used to build the model. That can be done by only choosing the most important attributes that improves the prediction accuracy. Another advantage of the feature selection process is reducing the resources (time and space) needed to build the model.

In this study, we used an automated R-based ML feature selection algorithm that ranks the attributes based on their Information Gain [28], which evaluates the importance of an attribute by measuring the entropy gain with respect to the outcome, and then ranks the attributes by their individual evaluations [27]. Only attributes that have information gain > 0 were subsequently used in building the machine learning models considered in this study.

### Machine learning classification models

Using the data of this study, we evaluated and compared six different classification techniques for predicting the Hypertension outcome: Artificial Neural Network (ANN), LogitBoost (LB), Locally Weighted Naive Bayes (LWB), Random Tree Forest (RTF), Sup- port Vector Machine (SVM) and Bayesian Network (BN).

**Artificial Neural Network (ANN)** [29] attempts to mimic the human brain to learn complex tasks. It is modeled as interconnected group of nodes in a way which is like the vast network of neurons in the human brain. Each of the network receives inputs from another source, combines them in some way, performs a generally nonlinear operation on the result and outputs the result. We train the Neural Networks with gradient descent back-propagation. We vary the number of hidden units {1, 2, 4, 8} and the momentum {0,0.2,0.5}.

**LogitBoost** (LB) [30] is a boosting algorithm that was originally developed to improve the classification performance of many weak classifiers. The LogitBoost classifier is based on **AdaBoost** procedure [30]. The adaBoost procedure trains the classifier on weighted versions of the training data and assigns higher weights for those training records that are misclassified. Such procedure is done for a sequence of weighted samples. Then the final classifier is defined to be a liner combination of the classifiers from each stage. LogiBoost uses an adaptive Newton algorithm to fit an adaptive multiple logistic regression model. LogiBoost is superior in handling noisy data.

**Locally Weighted Naive Bayes (LWB)** [31] is an instance-based learner that performs classification by comparing a test instance to a data set of pre-classified instances. The main assumption is that similar instances should have similar classifications. LWB is considered an enhancement of Naive Bayes where a linear regression model is fit to the data based on a weighting function centered on the instance for which a prediction is to be generated.

**Bayesian Network (BN)** [32] is a simple probabilistic classifier that is considered a generalization of the Naive Bayes classifier that removes the dependencies between variables. BN is designed for modeling under uncertainty where the nodes represent variables and arcs represent direct connections between them. BN model allows probabilistic beliefs about the variables to be updated automatically as new information becomes available. We used different search algorithms K2 [33], Hill Climbing [34], Repeated Hill Climber [35], LAGD Hill Climbing [36], TAN [37], Tabu search [38] and Simulated annealing [38].

**Support Vector Machine (SVM)** [39] represents the instances as a set of points of 2 types in *N* dimensional place and generates a (*N* − 1) dimensional hyperplane to separate those points into 2 groups. SVM attempts to find a straight line which separates those points into 2 types and is situated as far as possible from all those points. Training the SVM is done using Sequential Minimal Optimization algorithm [15]. We use Weka implementation of SMO [40]. We test SVM using polynomial, normalized polynomial, puk kernels and vary the complexity parameter {0.1, 10, and 30}. The value of the complexity parameter controls the tradeoff between fitting the training data and maximizing the separating margin.

**Random Tree Forest (RTF)** [41, 42] is a classification algorithm that works by forming multitude decision trees at training and at testing it outputs the class that is the mode of the classes (classification). Decision tree works by learning simple decision rules extracted from the data features. The deeper the tree, the more complex the decision rules and the fitter the model. Random decision forests overcome the problem of over fitting of the decision trees. All ML algorithms have been conducted using Weka Software (Version 3.8) (http://www.cs.waikato.ac.nz/ml/weka/) and R-based ML packages (Version 3.3.1) (https://www.r-project.org/).

### Model evaluation and validation

To evaluate our models, we used two main methods: the *hold out* [43] method and the 10-fold *cross-validation* method [44]. In principle, the main idea of the holdout method is to split the data into training set and test set. The training set is used by the classifier for the training process and the testing set is used to estimate the prediction error rate of the classifier after learning. For the holdout method, we have been using two data splits:

1. Training with 70% of the dataset and Testing with 30% of the dataset.
2. Training with 80% of the dataset and Testing with 20% of the dataset.

The main idea of the 10-fold cross validation is to partition the data set into 10 partitions. Each time one of the 10 partitions are used for testing the model and the other 9 partitions are used for training the model. So, each instance in the data set is used once in testing and 9 times in training. All results of the different metrics are then averaged to return the result. In general, the main advantage of the 10-fold cross-validation evaluation method is that it has a lower variance than a single hold-out set evaluator. It reduces this variance by averaging over 10 different partitions, therefore, it is less sensitive to any partitioning bias on the training or testing data.

In practice, the outcome of any binary classifier is one of the following four results:

- *True Positive* (TP) refers to the number of high risk patients who are classified as high risk.
- *False Negative* (FN) refers to the number of high risk patients who are classified as low risk patients.
- *False Positive* (FP) refers to the number of low risk patients who are classified as high-risk patients.
- *False Negative* (FN) refers to the number of low risk patients who are classified as low risk patients.

For all classifiers, the following evaluation metrics were calculated:

- **Sensitivity**: True Positive recognition rate
  $$\begin{array}{c} \text{Sensitivity}=\text{TP}/\left(\text{TP}+\text{FN}\right) \end{array}$$
- **Specificity**: True Negative recognition rate
  $$\begin{array}{c} \text{Specificity}=\text{TN}/\left(\text{TN}+\text{FP}\right) \end{array}$$
- **Precision**: It represents the percentage of tuples that the classifier has labeled as positive are actually positive
  $$\begin{array}{c} \text{Precision}=\text{TP}/\left(\text{TP}+\text{FP}\right) \end{array}$$
- **F-score**: It represents the harmonic mean of precision and sensitivity
  $$\begin{array}{c} \text{F-score}=2*\text{TP}/\left(2*\text{TP}+\text{FP}+\text{FN}\right) \end{array}$$
- **Root Mean Squared Error (RMSE)**: It is defined as the square root of the mean square error that measures the difference between values predicted by the model and the actual values observed, where *y*′ is a vector of *n* predictions and *y* is the vector of *n* observed (actual) values
- **Receiver Operating Characteristic (ROC) Curve**: It is a way to quantify the diagnostic value of a test over its whole range of possible cutoffs for classifying patients as positive vs. negative [45]. In each possible cutoff, the true positive rate and false positive rate is calculated as the *X* and *Y* coordinates in the ROC Curve.

## Results

**Fig 1:**
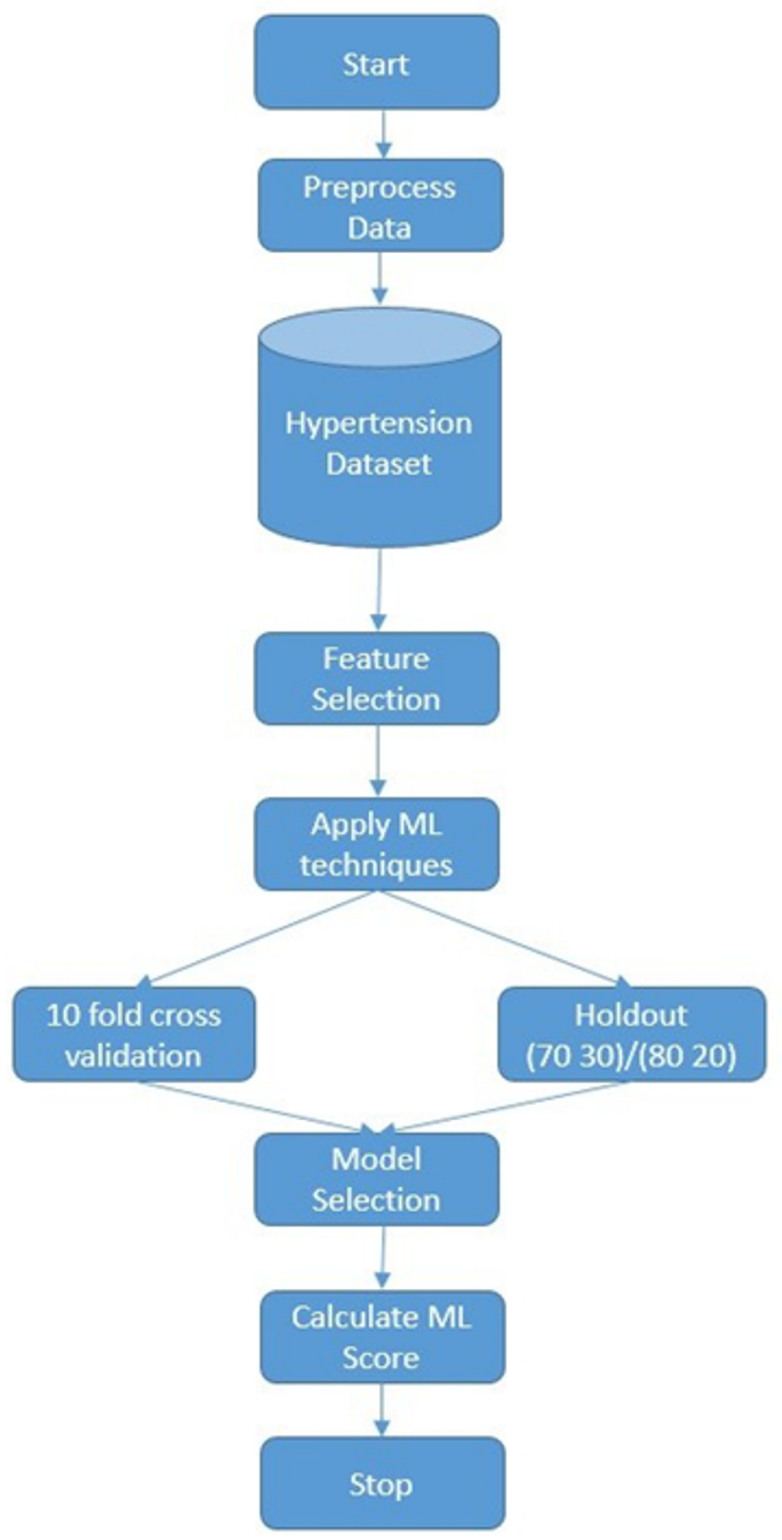
A flowchart of our experimental process.

Fig 1 illustrates the flowchart of the training and testing of the ML based techniques for predicting the risk of hypertension using the cardiorespiratory fitness data. First, dataset is preprocessed then SMOTE is applied on the dataset by creating synthetic examples of the class “*yes*” (patients experienced hypertension). The percentage of SMOTE instances created is 30%. Next, we apply the feature selection process where we rank the variables of the dataset according to their information gain and select the subset with the highest gain. Finally, we examine different machine learning models and evaluate their performance using the two main methods, hold out (70/30 and 80/20) and 10-fold cross-validation, based on different evaluation metrics.

**Fig 2:**
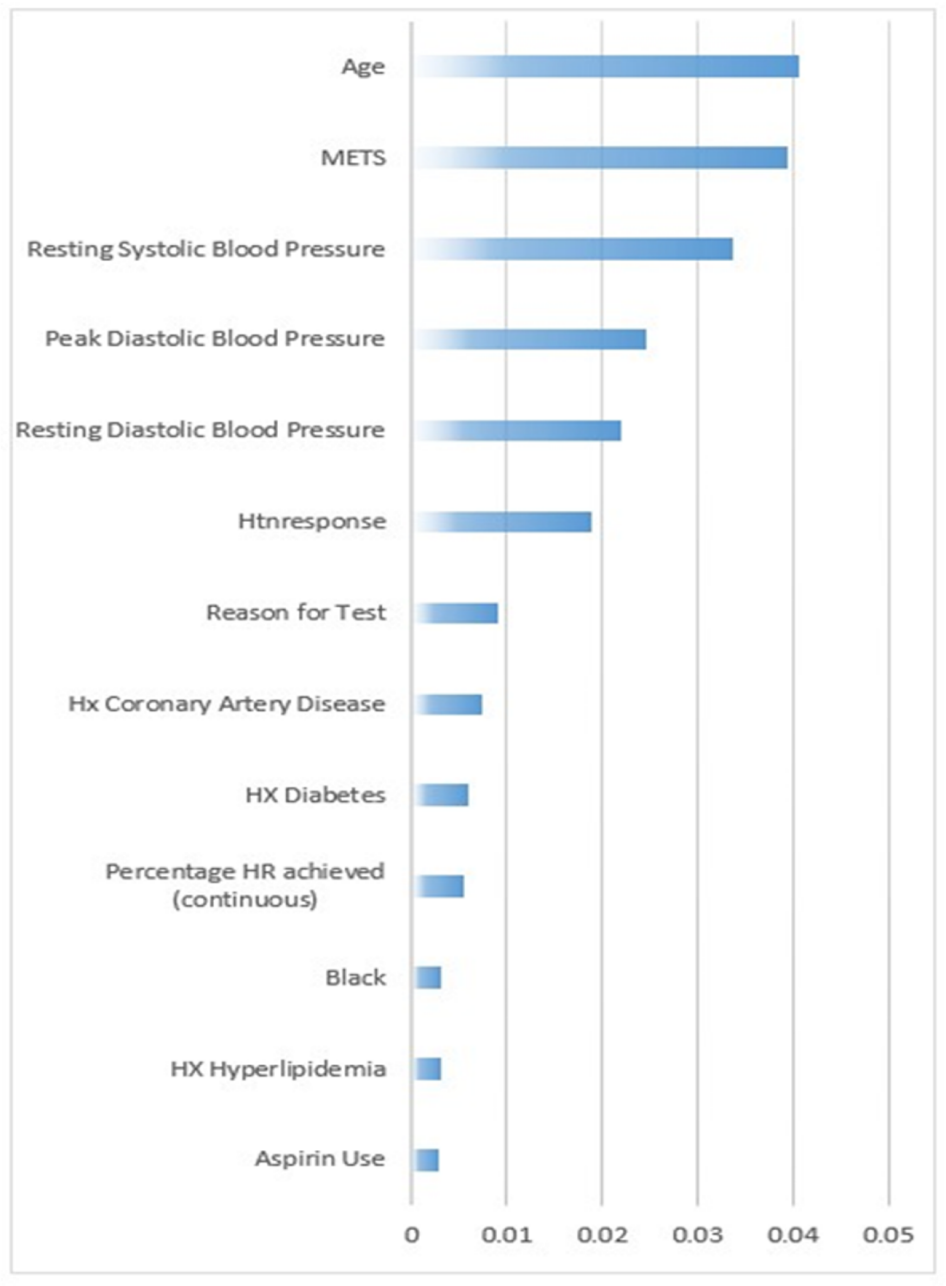
The information gain ranking of the attributes of the dataset.

As an outcome of the feature selection process, using the information gain ranking criteria, 13 attributes out of 49 were selected according to their information gain rank [28] for the Hypertension prediction. Age was the highest ranked feature for hypertension prediction. For our models, we selected the top ranked attributes that do not clinically contain collinear information: *Age*, *METS*, *Resting Systolic Blood Pressure*, *Peak Diastolic Blood Pressure*, *Resting Diastolic Blood Pressure*, *HX Coronary Artery Disease*, *Reason for test*, *History of Diabetes*, *Percentage HR achieved*, *Race*, *History of Hyperlipidemia*, *Aspirin Use*, *Hypertension response* (See Fig 2).

**Fig 3:**
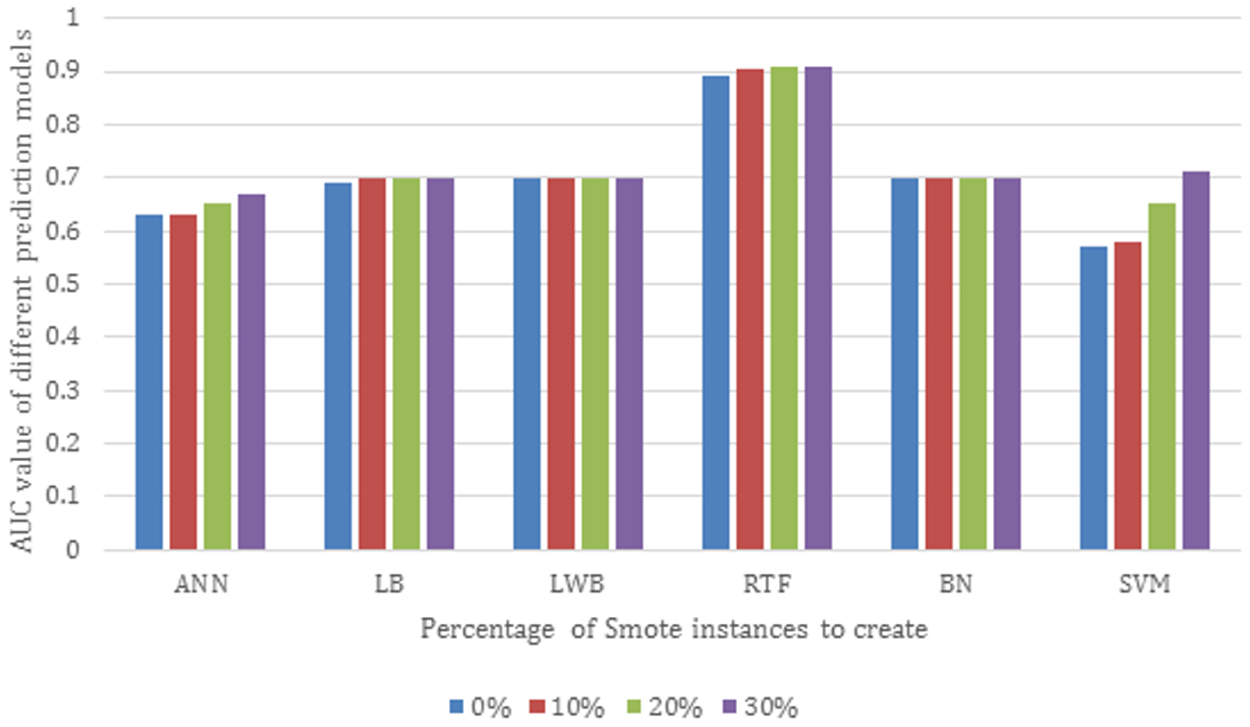
AUC of different models with different percentage of synthetic examples created using SMOTE evaluated using 10-fold cross validation.

We compared the impact of using SMOTE and Spread Subsample methods. We applied them with different percentage of synthetic examples. Fig 3 shows the area under the curve of six different models trained using LogitBoost (LB), Bayesian Network classifier (BN), Locally Weighted Naive Bayes (LWB), Artificial Neural Network (ANN), Support Vector Machine (SVM) and Random Tree Forest (RTF) with 0%, 10% and 30% of synthetic examples created using the SMOTE and evaluated using the 10-fold cross validation method. The results show that the performance of the RTF and SVM models using SMOTE has shown great improvement. The RTF and SVM achieve AUC of 0.91 and 0.71 respectively using the sampled dataset with 30% synthetic examples created in comparison to 0.9 and 0.57 respectively using the dataset without sampling. In contrast, the LWB and BN models show no improvement using SMOTE achieving both AUC of 0.7. LB and ANN models have shown a slight improvement using SMOTE by achieving AUC of 0.69 and 0.63 respectively without sampling and AUC of 0.7 and 0.67 using SMOTE with 30% created synthetic examples. In Spread Subsample technique, all the minority class instances (8015 instances) are used while some instances of the majority class are removed randomly until both classes are equally balanced.

**Fig 4:**
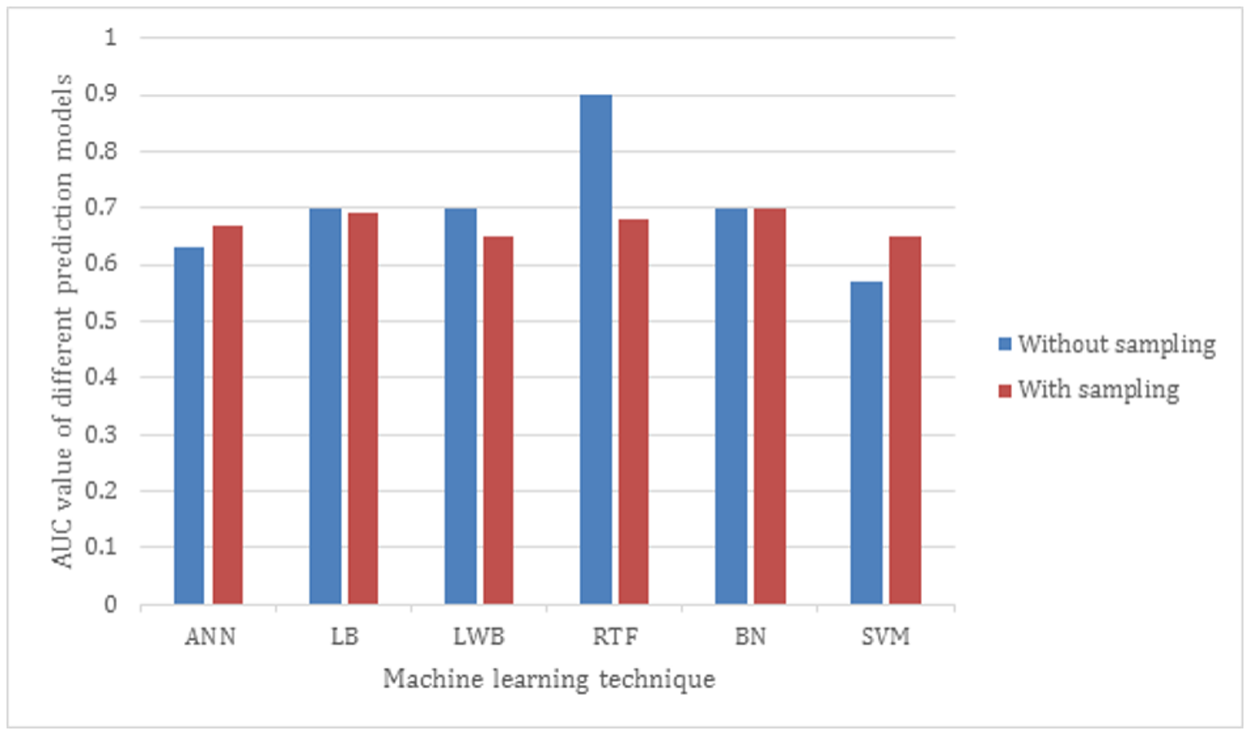
AUC of the different ML models using Spread Subsample technique.

Fig 4 presents the AUC of six different models trained using LB, BN, LWB, ANN, SVM and RTF. All models are evaluated using 10-fold cross validation. The results show that all models without using the Spread Subsample techniques outperforms the ones with sampling except for the SVM model. The RTF without sampling achieves 0.9 and dropped down dramatically to 0.68 using Spread Sampling. The SVM has shown a slight improvement using Spread Subsample by achieving AUC of 0.65 with sampling compared to 0.57 without sampling.

Table 2 presents the performance of the SVM using different kernels (polynomial kernel, normalized polynomial kernel and puk kernel) and complexity parameters (C) (0.1, 10 and 30) is tested and evaluated using 10-fold cross validation. The results show that the AUC increased as the complexity parameter increased up to 30. In addition, the SVM using puk kernel outperforms the SVM using other kernels achieving AUC of 0.71. The results show that using that using that puk kernel with complexity parameter equals 0.1 achieves the highest AUC of 0.59 evaluated using 10-fold cross validation.

**Table 2:** Comparison of the performance of Support Vector Machine (SVM) classifier with sampling using polynomial, normalized polynomial and puk kernels using complexity parameters 0.1, 10 and 30 using 10-fold cross validation using SMOTE.

	Polynomial			Normalized Polynomial			Puk		
	C = 0.1	C = 10	C = 30	C = 0.1	C = 10	C = 30	C = 0.1	C = 10	C = 30
**Sensitivity**	46.55	46.70	46.66	40.86	45.05	44.62	48.61	58.74	63.61
**Specificity**	77.39	77.31	77.33	79.17	77.40	78.09	78.44	78.90	78.97
**Precision**	52.57	52.56	52.57	51.36	51.76	52.30	54.83	59.97	61.95
**F-score**	49.38	49.45	49.44	45.51	48.18	48.15	51.53	59.35	62.77
**AUC**	0.62	0.62	0.62	0.60	0.61	0.61	0.64	0.69	0.71
**RMSE**	0.58	0.58	0.58	0.59	0.58	0.58	0.57	0.53	0.51

Table 3 presents the performance of Neural Networks with gradient descent back-propagation using hidden units H = {1, 2, 4, 8} and the momentum M = {0, 0.2, 0.5} using SMOTE evaluated using 10-fold cross validation. The number of hidden units and momentum rate that gives better AUC value is considered here. We achieve the highest AUC of 0.64 using H = 4 and M = 0. The performance of the Naïve Network Classifier using SMOTE evaluated using 10-fold cross validation is shown in Table 4. Seven different search algorithms (K2, Hill Climbing, Repeated Hill Climber, LAGD Hill Climbing, TAN, Tabu and Simulated Annealing) are evaluated as shown in Table 4. Bayesian Network classifier using Simulated Annealing algorithm achieves the highest AUC value of 0.70.

**Table 3:** Comparison of the performance of Artificial Neural Networks (ANN) classifier with gradient descent back-propagation using hidden units {1, 2, 4, 8} and the momentum {0,0.2, 0.5} using 10-fold cross validation using SMOTE.

	H = 1			H = 2			H = 4			H = 8		
	M = 0	M = 0.2	M = 0.5	M = 0	M = 0.2	M = 0.5	M = 0	M = 0.2	M = 0.5	M = 0	M = 0.2	M = 0.5
**Sensitivity**	59.30	50.06	51.44	49.77	51.33	42.21	30.06	32.29	62.05	51.38	56.41	37.49
**Specificity**	64.34	73.22	71.68	72.45	71.52	79.07	88.00	85.04	57.74	73.50	66.83	82.34
**Precision**	47.24	50.16	49.44	49.30	49.25	52.05	57.43	53.75	44.15	51.07	47.79	53.33
**F-score**	52.59	50.11	50.42	49.53	50.27	46.61	39.46	40.34	51.59	51.22	51.74	44.03
**AUC**	0.66	0.66	0.65	0.66	0.66	0.67	0.67	0.67	0.66	0.67	0.67	0.67
**RMSE**	0.47	0.47	0.47	0.47	0.47	0.46	0.46	0.46	0.47	0.47	0.47	0.47

**Table 4:** Comparison of the performance of Bayesian Network classifier (BN) using different search algorithms K2, Hill Climbing, Repeated Hill Climber, LAGD Hill Climbing, TAN, Tabu and Simulated Annealing using 10-fold cross validation using SMOTE.

	K2	Hill Climbing	Repeated Hill Climber	LAGD Hill Climbing	TAN	Tabu	Simulated Annealing
**Sensitivity**	45.29	45.24	45.24	33.49	40.42	44.95	36.26
**Specificity**	79.91	79.90	79.90	85.01	83.36	79.94	85.86
**Precision**	54.83	54.79	54.79	54.60	56.67	54.68	57.99
**F-score**	49.60	49.56	49.56	41.51	47.19	49.34	44.62
**AUC**	0.70	0.70	0.70	0.67	0.70	0.70	0.70
**RMSE**	0.47	0.47	0.47	0.46	0.45	0.47	0.45

**Fig 5:**
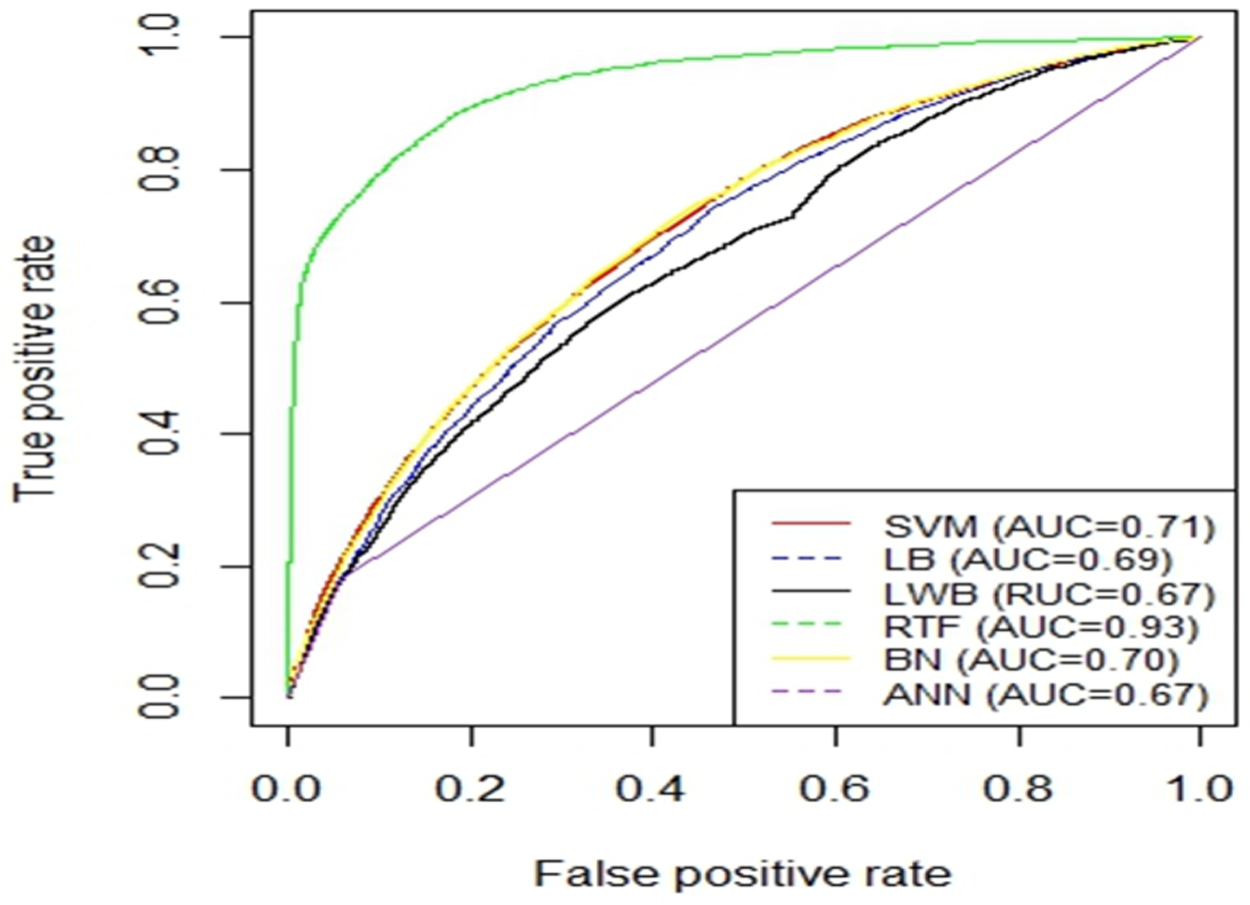
AUC Curves for the Different Machine Learning Models using SMOTE evaluated using 10-fold cross-validation.

**Fig 6:**
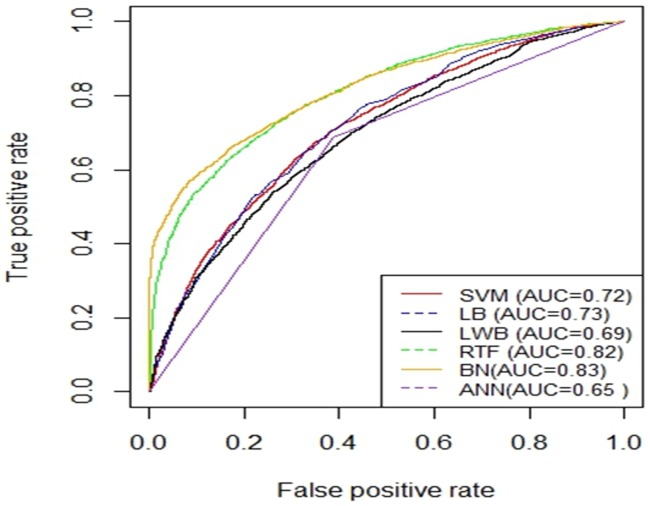
AUC Curves for the Different Machine Learning Models using SMOTE and evaluated using holdout (70/30).

**Fig 7:**
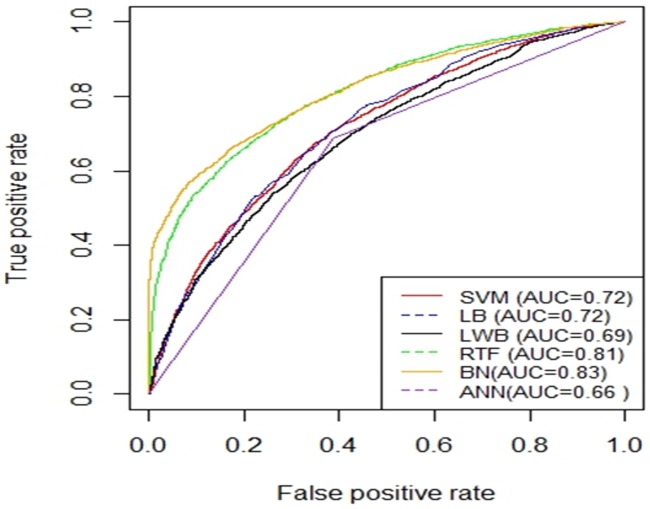
AUC Curves for the Different Machine Learning Models using SMOTE and evaluated using holdout (80/20).

Fig 5 shows the AUC curves of the different models using the balanced dataset which were generated using SMOTE and validated using 10-fold cross-validation method. Figs 6 and 7 show the ROC curves of the different models using the balanced dataset which were generated using SMOTE and validated using two splits of the holdout methods: 70/30 and 80/20, respectively. Among the different evaluation methods, the Random Tree Forest (RTF) model achieves the highest AUC using the 10-fold cross-validation method (0.93), holdout method 70/30 (0.83) and holdout method 80/20 (0.88).

Table 5 summarizes the performance of the different machine learning techniques on sampled data using SMOTE using 10-fold cross validation while Tables 6 and 7 summarize the performance of the different models on sampled data using SMOTE evaluated using the two splits of the holdout methods 70/30 and 80/20, respectively. For each metric (row) in the tables, we highlighted the highest value in bold font and underlined the lowest value.

**Table 5:** The performance of the Different Machine Learning Models evaluated using the 10-fold cross validation method using SMOTE. The RTF model achieves the highest AUC (0.93), F-Score (86.70%), sensitivity (69,96%) and Specificity (91.71%).

	ANN	LB	LWB	RTF	BN	SVM
**Sensitivity**	30.06%	31.28%	37.22%	69.96%	36.26%	63.61%
**Specificity**	88.00%	88.56%	84.05%	91.71%	85.86%	78.97%
**Precision**	57.43%	59.53%	55.67%	81.69%	57.99%	61.95%
**F-score**	39.46%	41.01%	53%	86.70%	44.62%	62.77%
**AUC**	0.67	0.69	0.67	0.93	0.70	0.71
**RMSE**	0.46	0.54	0.46	0.34	0.45	0.51

**Table 6:** The performance of the Different Machine Learning Models evaluated using the Hold Out method (70/30) using SMOTE. The RTF model achieve the highest AUC (0.88), Sensitivity (74.30%), Precision (73.50%) and F-Score (73.90%).

	ANN	LB	LWB	RTF	BN	SVM
**Sensitivity**	39.50%	31.40%	40.80%	74.30%	48.80%	26.30%
**Specificity**	86.50%	88.60%	81.80%	85.60%	79.30%	88.60%
**Precision**	61.20%	59.80%	54.60%	73.50%	55.90%	55.50%
**F-score**	48%	41.20%	46.64%	73.90%	52.10%	35.70%
**AUC**	0.72	0.70	0.70	0.88	0.71	0.58
**RMSE**	0.54	0.451	0.46	0.36	0.47	0.58

**Table 7:** The performance of the Different Machine Learning Models evaluated using the Hold Out method (80/20) using SMOTE. The RTF model achieves the highest AUC (0.89), Sensitivity (75%), Precision (73%) and F-Score (74%). The SVM model achieves the highest Specificity (88.9%).

	ANN	LB	LWB	RTF	BN	SVM
**Sensitivity**	40%	31.3%	43%	75%	49.5%	28.2%
**Specificity**	88.4%	88.5%	80.92%	86.2%	79.8%	88.9%
**Precision**	65.2%	59.3%	54.8%	73%	56.8%	57.7%
**F-score**	49.8%	40.9%	48.23%	74%	52.9%	37.9%
**AUC**	0.74	0.7	0.7	0.89	0.72	0.59
**RMSE**	0.44	0.45	0.46	0.46	0.42	0.57

We have evaluated the different models using different methods and various evaluation metrics. In general, the Random Tree Forest (RTF) model significantly outperformed all other models for the Specificity (91.7%), Precision (81.69%), F-score (86.7%), AUC (0.93) and Root Mean Squared Error (0.34) metrics evaluated using 10-fold cross validation.

- **Sensitivity metric**: The ANN and LB showed very comparable performance and came in the second place by achieving 30.06% and 31.28% respectively.
- **Specificity metric**: The LB and ANN showed a very comparable performance and came in the first place achieving 88.56% and 88% respectively. The SVM showed the worst performance (78.97%).
- **Precision metric**: The RTF has the highest precision of 81.69% followed by the SVM achieving 61.95%. The ANN and BN have comparable performance an achieving 57.43% and 57% respectively. The LWB showed the worst performance (55.67%).
- **F-score**: The RTF took first place by achieving 86.70% while ANN came at the last place at only 39.46%. The LB has the highest RMSE of 0.54.

The two data splits (70/30 and 80/20) of the hold out evaluation method showed different and comparable results from the 10-fold cross-validation method. For both data splits, the Random Tree Forest (RTF) showed the best performance of the Sensitivity, Specificity, Precision, F-score, AUC and Root Mean Squared Error metrics. For the two data splits, The BN showed the lowest performance for the Specificity metric. The Support Vector Machine (SVM) showed the lowest performance for the F-score, Sensitivity and Root Mean Squared Error metrics, for both data splits. The LWB showed the lowest performance for the Precision metric. In general, for all metrics, the results show that it is not necessarily that complex machine learning models such as Support Vector Machine (SVM) and Artificial Neural Networks (ANN) can always outperform simpler models such as the Random Tree Forest (RTF) model and the Bayesian Network classifier (BN) [46].

In principle, parametric models (e.g., SVM, ANN) tend to perform well in high-dimensioned classification problems that may have over hundreds of thousands of dimensions, which is not the case in this study. In addition, such models do not tend to perform well if the classes of the problem are strongly overlapping. In such cases, they can suffer from remembering local groupings as by their nature they summarize information in a way. ANN can usually outperform other methods if the dataset is very large and if the structure of the data is complex (e.g., they have many layers) [46]. On the other hand, Random Forest can be considered as an ensembling techniques which uses fully grown decision trees and combines them in a way that improve the accuracy of predictions by reducing variance. In addition, it inherently contains some underlying decision trees that omit the noise generating variable/feature(s). The Bayesian network classifier (BN) has the advantages of using very simple assumptions about the independence of the variables and shows superior performance in capturing interactions among input variables [47, 48].

## Discussion

Several studies have been conducted for predicting the risk of hypertension using statistical and machine learning techniques [49–52]. Samant and Rao presented a Levenberg-Marquardt back-propagation neural network model to predict hypertension. The dataset used was collected over 10 years at the Hemorheology Laboratory of the Indian Institute of Technology Bombay (IITB) hospital in Mumbai, India. The predictors used in building the model are blood pressure, serum proteins, albumin, hematocrit, cholesterol, triglycerides, and hemorheological parameters. The authors evaluated the performance of the model using different number of nodes in the hidden layer and they concluded that using 20 nodes in the first hidden layer and 5 nodes in the second hidden layer achieves the best accuracy of 92.85%.

Ture et al. [53] reported about the performance of four statistical models and two artificial neural networks models on predicting the risk of hypertension using a dataset consisting of 694 records. The predictors used in building the models are age, sex, family history, smoking habits, lipoproteins, triglycerides, uric acid, cholesterol, and BMI. Based on the sensitivity and specificity analysis of the models, the study shows that the artificial neural networks model based on Radial Basis Function [45] outperforms all the models achieving sensitivity of 95.20% and specificity of 66.70%.

Al-Nozha et al. [54] determined the prevalence of hypertension among Saudis of both gender aged between 30 to 70 years in rural communities over the period between 1995 and 2000 using a dataset of 17,230 records. They proposed a predictive model for hypertension using Logistic regression. The prevalence of hypertension in males and females were 28.6% and 23.9% respectively. Predictive models for hypertension using support vector machine (SVM) using several kernel functions were compared in [55]. Three medical datasets of size 6000 were used and collected from the Department of Health Examination from those seeking an annual physical health check-up at Chang Gung Memorial Hospital in Tao-Yuan, Taiwan). In addition to nine datasets from the UCI repository [56] (census income, shuttle, mushroom, letter, ionosphere, vehicle silhouettes, spambase, vowel, and sonar), the largest dataset among these datasets is Census income and consists of 32,561 records. Results show that the SVM using multiplication kernel outperforms other approaches archiving average accuracy of 84.29% and 93.39% using large datasets (greater than 5000 records) and small datasets (fewer than 5000 records) respectively.

This study is designed to take advantage of the unique and rich clinical research dataset consisting of 23,095 patients, collected by the FIT project to investigate the relative performance of different machine learning classification techniques for predicting the individuals at risk of developing hypertension using medical records of cardiorespiratory fitness. To the best of our knowledge, this is the first study that compares the performance of six different ML models for predicting the individuals at risk of developing hypertension using cardiorespiratory fitness data. Using different validation methods, the RTF model on our dataset has shown the best performance (AUC = 0.93) which outperforms the models of the previous studies.

## Conclusion

Machine learning techniques have been shown to provide solid prediction capabilities in various application domains including medicine and healthcare [18, 22]. In this study, we presented an evaluation and comparison of six popular machine learning techniques on predicting the patients who could be at risk of developing hypertension using medical records of Cardiorespiratory Fitness from the Henry Ford Testing (FIT) Project. The results show that it is not necessarily that the more complex the machine learning model, the better prediction accuracy that can be achieved. Simpler models can perform better in some cases as well. The results have also shown that it is critical to carefully explore and evaluate the performance of the machine learning models using various model evaluation methods as the prediction accuracy can significantly differ. These results confirm the explorative nature of the machine learning process that requires iterative and explorative experiments in order to discover the model design that can achieve the target accuracy for a specific problem.

## Ethical approval

The FIT project is approved by the IRB (ethics committee) of Henry Ford Hospital (IRB #: 5812).

## References

[1] Jeppesen J, Hein HO, Suadicani P, Gyntelberg F. High triglycerides and low HDL cholesterol and blood pressure and risk of ischemic heart disease. Hypertension. 2000;36(2):226–232. doi: 10.1161/01.HYP.36.2.226 10948082 10.1161/01.hyp.36.2.226

[2] Whelton PK, Carey RM, Aronow WS, Casey DE, Collins KJ, Dennison Himmelfarb C, et al 2017 ACC/AHA/AAPA/ABC/ACPM/AGS/APhA/ASH/ASPC/NMA/PCNA Guideline for the Prevention, Detection, Evaluation, and Management of High Blood Pressure in Adults. Journal of the American College of Cardiology. 2017 doi: 10.1016/j.jacc.2017.11.005 10.1016/j.jacc.2017.11.006 29146535

[3] Organization WH, et al. Clinical guidelines for the management of hypertension; 2005.

[4] Chowdhury R, Kunutsor S, Vitezova A, Oliver-Williams C, Chowdhury S, Kiefte-de Jong JC, et al Vitamin D and risk of cause specific death: systematic review and meta-analysis of observational cohort and randomised intervention studies. Bmj. 2014;348:g1903 doi: 10.1136/bmj.g1903 24690623 10.1136/bmj.g1903 PMC3972416

[5] Carson JL, Sieber F, Cook DR, Hoover DR, Noveck H, Chaitman BR, et al Liberal versus restrictive blood transfusion strategy: 3-year survival and cause of death results from the FOCUS randomised controlled trial. The Lancet. 2015;385(9974):1183–1189. doi: 10.1016/S0140-6736(14)62286-8 10.1016/S0140-6736(14)62286-8 PMC4498804 25499165

[6] Kung HC, Xu J. Hypertension-related Mortality in the United States, 2000-2013. NCHS data brief. 2015;(193):1–8. 25932893

[7] Al-Mallah MH, Keteyian SJ, Brawner CA, Whelton S, Blaha MJ. Rationale and design of the Henry Ford Exercise Testing Project (the FIT project). Clinical cardiology. 2014;37(8):456–461. doi: 10.1002/clc.22302 25138770 10.1002/clc.22302 PMC6649579

[8] Bruce R, Kusumi F, Hosmer D. Maximal oxygen intake and nomographic assessment of functional aerobic impairment in cardiovascular disease. American heart journal. 1973;85(4):546–562. doi: 10.1016/0002-8703(73)90502-4 4632004 10.1016/0002-8703(73)90502-4

[9] Members C, Gibbons RJ, Balady GJ, Bricker JT, Chaitman BR, Fletcher GF, et al Journal of the American College of Cardiology. 2002;40(8):1531–1540. doi: 10.1016/S0735-1097(02)02164-2 12392846 10.1016/s0735-1097(02)02164-2

[10] Juraschek SP, Blaha MJ, Whelton SP, Blumenthal R, Jones SR, Keteyian SJ, et al Physical fitness and hypertension in a population at risk for cardiovascular disease: the Henry Ford ExercIse Testing (FIT) Project. Journal of the American Heart Association. 2014;3(6):e001268 doi: 10.1161/JAHA.114.001268 25520327 10.1161/JAHA.114.001268 PMC4338714

[11] Hung RK, Al-Mallah MH, McEvoy JW, Whelton SP, Blumenthal RS, Nasir K, et al Prognostic value of exercise capacity in patients with coronary artery disease: the FIT (Henry Ford ExercIse Testing) project In: Mayo Clinic Proceedings. vol. 89 Elsevier; 2014 p. 1644–1654. 25440889 10.1016/j.mayocp.2014.07.011

[12] Juraschek SP, Blaha MJ, Blumenthal RS, Brawner C, Qureshi W, Keteyian SJ, et al Cardiorespiratory fitness and incident diabetes: the FIT (Henry Ford ExercIse Testing) project. Diabetes Care. 2015;38(6):1075–1081. doi: 10.2337/dc14-2714 25765356 10.2337/dc14-2714

[13] Qureshi WT, Alirhayim Z, Blaha MJ, Juraschek SP, Keteyian SJ, Brawner CA, et al Cardiorespiratory fitness and risk of incident atrial fibrillation: results from the Henry Ford ExercIse Tesing (FIT) project. Circulation. 2015; p. CIRCULATIONAHA–114. doi: 10.1161/CIRCULATIONAHA.115.018758 10.1161/CIRCULATIONAHA.114.014833 25904645

[14] Alpaydin E. Introduction to machine learning. MIT press; 2014.

[15] Marsland S. Machine learning: an algorithmic perspective. CRC press; 2015.

[16] Aggarwal CC. Data classification: algorithms and applications. CRC Press; 2014.

[17] John Walker S. Big data: A revolution that will transform how we live, work, and think; 2014.

[18] Waljee AK, Higgins PD. Machine learning in medicine: a primer for physicians. The American journal of gastroenterology. 2010;105(6):1224 doi: 10.1038/ajg.2010.173 20523307 10.1038/ajg.2010.173

[19] Kayyali B, Knott D, Van Kuiken S. The big-data revolution in US health care: Accelerating value and innovation. Mc Kinsey & Company. 2013;2(8):1–13.

[20] Raghupathi W, Raghupathi V. Big data analytics in healthcare: promise and potential. Health information science and systems. 2014;2(1):3 doi: 10.1186/2047-2501-2-3 25825667 10.1186/2047-2501-2-3 PMC4341817

[21] Sakr S, Elshawi R, Ahmed AM, Qureshi WT, Brawner CA, Keteyian SJ, et al Comparison of machine learning techniques to predict all-cause mortality using fitness data: the Henry ford exercIse testing (FIT) project. BMC medical informatics and decision making. 2017;17(1):174 doi: 10.1186/s12911-017-0566-6 29258510 10.1186/s12911-017-0566-6 PMC5735871

[22] Alghamdi M, Al-Mallah M, Keteyian S, Brawner C, Ehrman J, Sakr S. Predicting diabetes mellitus using SMOTE and ensemble machine learning approach: The Henry Ford ExercIse Testing (FIT) project. PLoS One. 2017;12(7):e0179805 doi: 10.1371/journal.pone.0179805 28738059 10.1371/journal.pone.0179805 PMC5524285

[23] Kurgan L, Cios KJ. Discretization algorithm that uses class-attribute interdependence maximization. In: Proceedings of the 2001 International Conference on Artificial Intelligence (IC-AI 2001); 2001. p. 980–987.

[24] Chawla NV. Data mining for imbalanced datasets: An overview In: Data mining and knowledge discovery handbook. Springer; 2009 p. 875–886.

[25] Kubat M, Matwin S, et al. Addressing the curse of imbalanced training sets: one-sided selection. In: ICML. vol. 97. Nashville, USA; 1997. p. 179–186.

[26] Chawla NV, Bowyer KW, Hall LO, Kegelmeyer WP. SMOTE: synthetic minority over-sampling technique. Journal of artificial intelligence research. 2002;16:321–357.

[27] Guyon I, Elisseeff A. An introduction to variable and feature selection. Journal of machine learning research. 2003;3(Mar):1157–1182.

[28] Kent JT. Information gain and a general measure of correlation. Biometrika. 1983;70(1):163–173. doi: 10.1093/biomet/70.1.163

[29] Arbib MA. The handbook of brain theory and neural networks. MIT press; 2003.

[30] Friedman J, Hastie T, Tibshirani R, et al Additive logistic regression: a statistical view of boosting (with discussion and a rejoinder by the authors). The annals of statistics. 2000;28(2):337–407.

[31] Frank E, Hall M, Pfahringer B. Locally weighted naive bayes. In: Proceedings of the Nineteenth conference on Uncertainty in Artificial Intelligence. Morgan Kaufmann Publishers Inc.; 2002. p. 249–256.

[32] Friedman N, Geiger D, Goldszmidt M. Bayesian network classifiers. Machine learning. 1997;29(2-3):131–163. doi: 10.1023/A:1007465528199

[33] Cooper GF, Herskovits E. A Bayesian method for the induction of probabilistic networks from data. Machine learning. 1992;9(4):309–347. doi: 10.1007/BF00994110

[34] Buntine W. A guide to the literature on learning probabilistic networks from data. IEEE Transactions on knowledge and data engineering. 1996;8(2):195–210.

[35] Yuret D, De La Maza M. Dynamic hill climbing: Overcoming the limitations of optimization techniques. In: The Second Turkish Symposium on Artificial Intelligence and Neural Networks; 1993. p. 208–212.

[36] Abramovici M, Neubach M, Fathi M, Holland A. Competing fusion for bayesian applications. In: Proceedings of IPMU. vol. 8; 2008. p. 379.

[37] Cheng J, Greiner R. Comparing Bayesian network classifiers. In: Proceedings of the Fifteenth conference on Uncertainty in artificial intelligence. Morgan Kaufmann Publishers Inc.; 1999. p. 101–108.

[38] Bouckaert RR. Bayesian belief networks: from construction to inference; 2001.

[39] Hearst MA, Dumais ST, Osuna E, Platt J, Scholkopf B. Support vector machines. IEEE Intelligent Systems and their applications. 1998;13(4):18–28.

[40] Zeng ZQ, Yu HB, Xu HR, Xie YQ, Gao J. Fast training Support Vector Machines using parallel sequential minimal optimization. In: Intelligent System and Knowledge Engineering, 2008. ISKE 2008. 3rd International Conference on. vol. 1. IEEE; 2008. p. 997–1001.

[41] Ho TK. Random decision forests. In: Document Analysis and Recognition, 1995., Proceedings of the Third International Conference on. vol. 1. IEEE; 1995. p. 278–282.

[42] Prasad AM, Iverson LR, Liaw A. Newer classification and regression tree techniques: bagging and random forests for ecological prediction. Ecosystems. 2006;9(2):181–199. doi: 10.1007/s10021-005-0054-1

[43] Hawkins DM. The problem of overfitting. Journal of chemical information and computer sciences. 2004;44(1):1–12. doi: 10.1021/ci0342472 14741005 10.1021/ci0342472

[44] Ross K, Jensen C, Snodgrass R, Dyreson C, Jensen C, Snodgrass R, et al. Cross-Validation. Encyclopedia of Database Systems; 2009.

[45] Haykin S. Neural networks: a comprehensive foundation. Prentice Hall PTR; 1994.

[46] Caruana R, Niculescu-Mizil A. An empirical comparison of supervised learning algorithms. In: Proceedings of the 23rd international conference on Machine learning. ACM; 2006. p. 161–168.

[47] Lee SM, Abbott PA. Bayesian networks for knowledge discovery in large datasets: basics for nurse researchers. Journal of biomedical informatics. 2003;36(4):389–399. doi: 10.1016/j.jbi.2003.09.022 14643735 10.1016/j.jbi.2003.09.022

[48] Heckerman D, Geiger D, Chickering DM. Learning Bayesian networks: The combination of knowledge and statistical data. Machine learning. 1995;20(3):197–243. doi: 10.1023/A:1022623210503

[49] Echouffo-Tcheugui JB, Batty GD, Kivimäki M, Kengne AP. Risk models to predict hypertension: a systematic review. PloS one. 2013;8(7):e67370 doi: 10.1371/journal.pone.0067370 23861760 10.1371/journal.pone.0067370 PMC3702558

[50] Poli R, Cagnoni S, Livi R, Coppini G, Valli G. A neural network expert system for diagnosing and treating hypertension. Computer. 1991;24(3):64–71.

[51] Samant R, Rao S. Evaluation of artificial neural networks in prediction of essential hypertension. International Journal of Computer Applications. 2013;81(12). doi: 10.5120/14067-2331

[52] Abdullah AA, Zakaria Z, Mohamad NF. Design and development of fuzzy expert system for diagnosis of hypertension. In: Intelligent Systems, Modelling and Simulation (ISMS), 2011 Second International Conference on. IEEE; 2011. p. 113–117.

[53] Ture M, Kurt I, Kurum AT, Ozdamar K. Comparing classification techniques for predicting essential hypertension. Expert Systems with Applications. 2005;29(3):583–588. doi: 10.1016/j.eswa.2005.04.014

[54] Al-Nozha MM, Abdullah M, Arafah MR, Khalil MZ, Khan NB, Al-Mazrou YY, et al Hypertension in Saudi Arabia. Saudi medical journal. 2007;28(1):77 17206295

[55] Su CT, Yang CH. Feature selection for the SVM: An application to hypertension diagnosis. Expert Systems with Applications. 2008;34(1):754–763. doi: 10.1016/j.eswa.2006.10.010

[56] Blake CL, Merz CJ. UCI Repository of machine learning databases [http://www.ics.uci.edu/~mlearn/MLRepository.html]. Irvine, CA: University of California. Department of Information and Computer Science. 1998;55.

